# Supplementary material for: Mechanisms of Binding and Immune Escape Resistance for Broadly Neutralizing Antibodies Targeting Distinct Conserved SARS-CoV-2 Spike Epitopes: A Hierarchical Approach Integrating Mutational Profiling and Energy Landscape Analysis
Source: Int J Mol Sci. 2026 Apr 30;27(9):4025. doi: 10.3390/ijms27094025 (PMC13163605; doi:10.3390/ijms27094025)
Supplement: Supplementary file 1 [file ijms-27-04025-s001.zip › ijms-4268164-supplementary.pdf]

Supplementary Material

Mechanisms of Binding and Immune Escape Resistance for Broadly Neutralizing Antibodies Targeting Distinct Conserved SARS-CoV-2 Spike Epitopes: A Hierarchical Approach Integrating Mutational Profiling and Energy Landscape Analysis

Mohammed Alshahrani,[1] Will Gatlin[1], Max Ludwick[1], Lucas Turano[1], Brandon Foley,[1] Gennady Verkhivker [1,2,3]*

[1] Keck Center for Science and Engineering, Graduate Program in Computational and Data Sciences, Schmid College of Science and Technology, Chapman University, Orange, CA 92866, United States of America
alshahrani@chapman.edu (M.A); wgatlin@chapman.edu (W.G.); ludwick@chapman.edu (M.L.); turano@chapman.edu (L.T.); brfoley@chapaman.edu (B.F.); verkhivk@chapman.edu (G.V)

[2] Department of Biomedical and Pharmaceutical Sciences, Chapman University School of Pharmacy, Irvine, CA 92618, United States of America
[3]Department of Pharmacology, Skaggs School of Pharmacy and Pharmaceutical Sciences, University of California San Diego, 9500 Gilman Drive, La Jolla, CA 92093, United States of America

* Correspondence: verkhivk@chapman.edu; Tel.: +1-714-516-4586 (G.V)

S309 (PDB 7YAD)

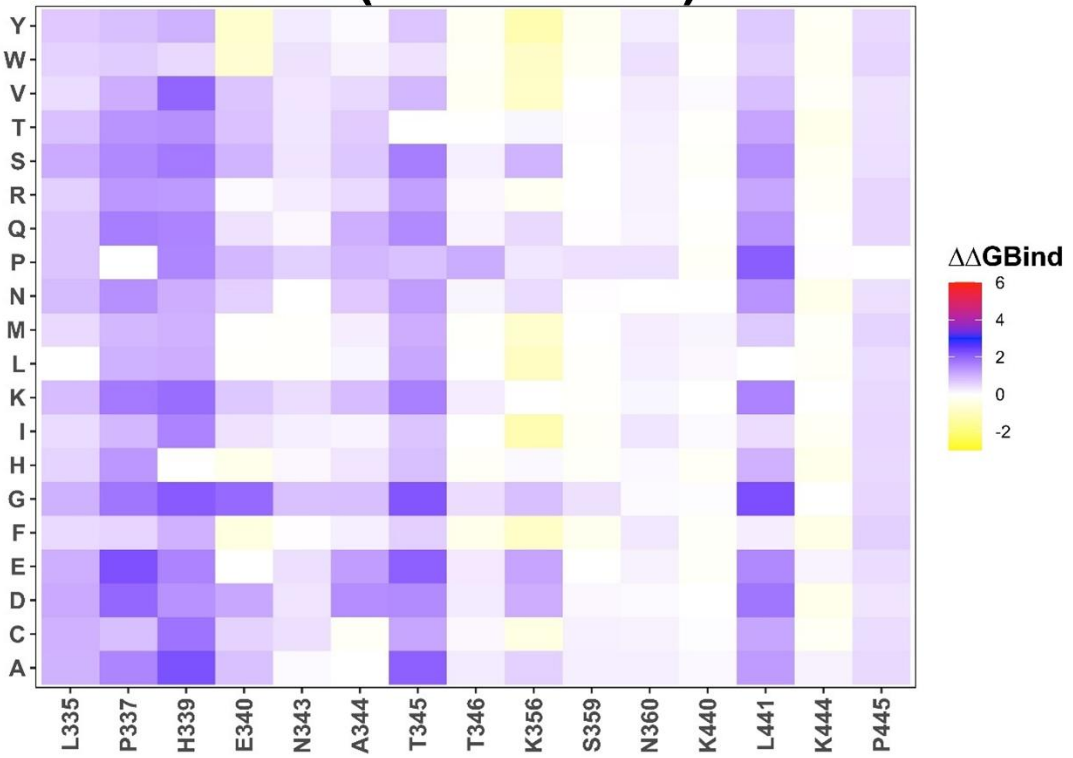

SA58 (PDB 7Y0W)

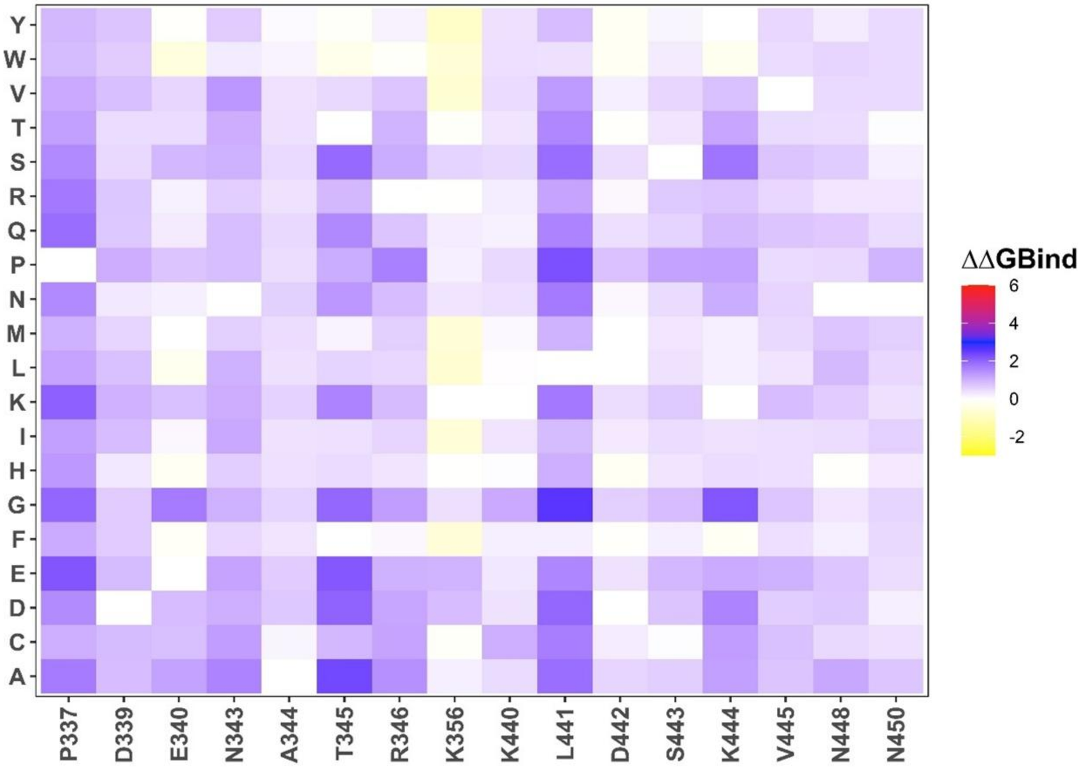

Figure S1. Mutational scanning of binding for the RBD complexes with SCORE-A epitope class S309 and SA58 antibodies. The mutational scanning heatmaps for the binding epitope residues in the S-RBD complexes with S309 (top panel) and SA58 (bottom panel). The heatmaps show the computed binding free energy changes for 20 single mutations on the sites of variants. The squares on the heatmap are colored using a 4-colored scale blue-white-yellow-red, with blue indicating the largest unfavorable effect on binding and stability, while yellow-red points to mutations that have favorable effect and improve binding.

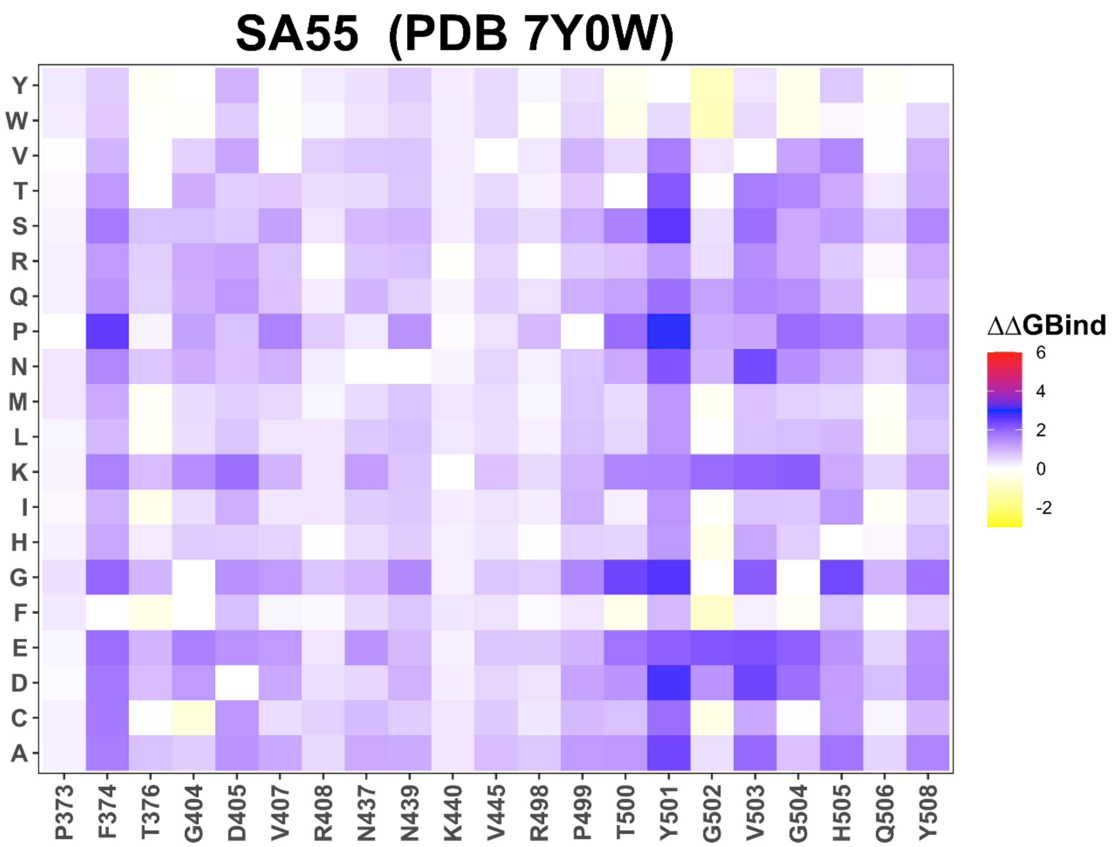

Figure S2. Mutational scanning of binding for the RBD complexes with SCORE-B epitope class SA55 antibody. The mutational scanning heatmaps for the binding epitope residues in the S-RBD complex with SA55 antibody. The heatmaps show the computed binding free energy changes for 20 single mutations on the sites of variants. The squares on the heatmap are colored using a 4-colored scale blue-white-yellow-red, with blue indicating the largest unfavorable effect on binding and stability, while yellow-red points to mutations that have favorable effect and improve binding.

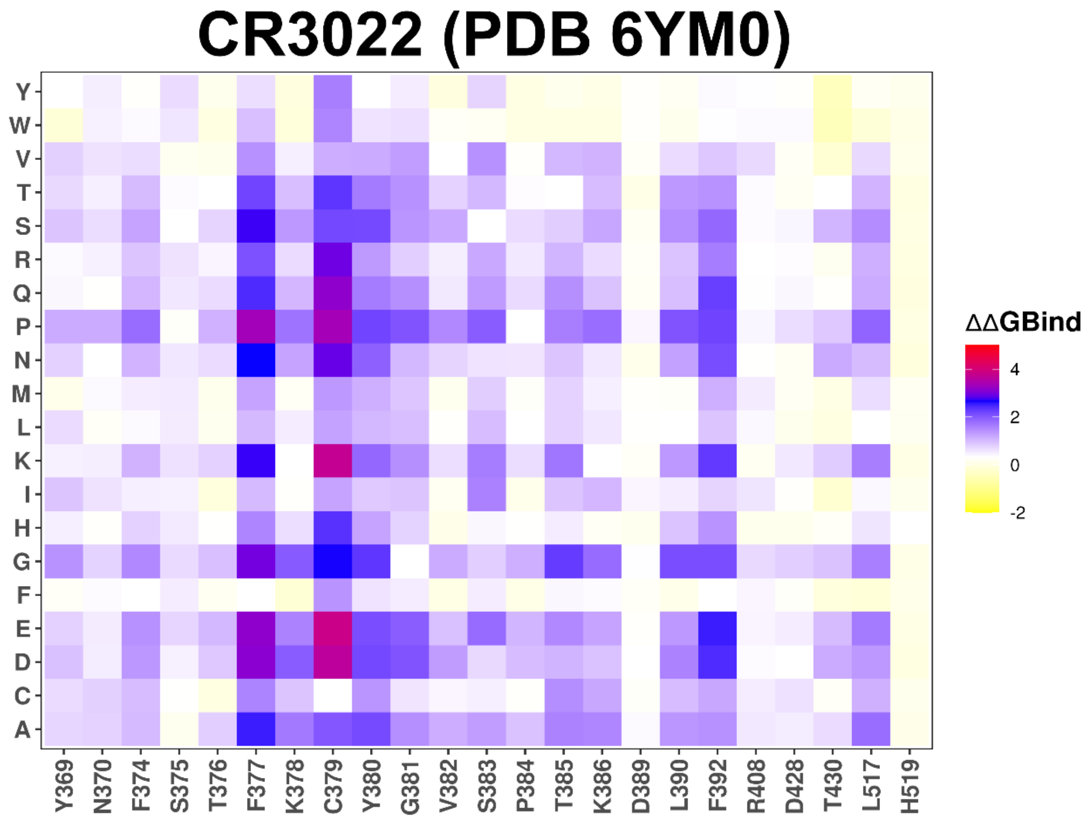
CR3022 (PDB 6YM0)
Y
W
V
T
S
R
Q
P
N
M
L
K
I
H
G
F
E
D
C
A
Y369
N370
F374
S375
T376
F377
K378
C379
Y380
G381
V382
S383
P384
T385
K386
D389
L390
F392
R408
D428
T430
L517
H519
ΔΔGBind
4
2
0
-2

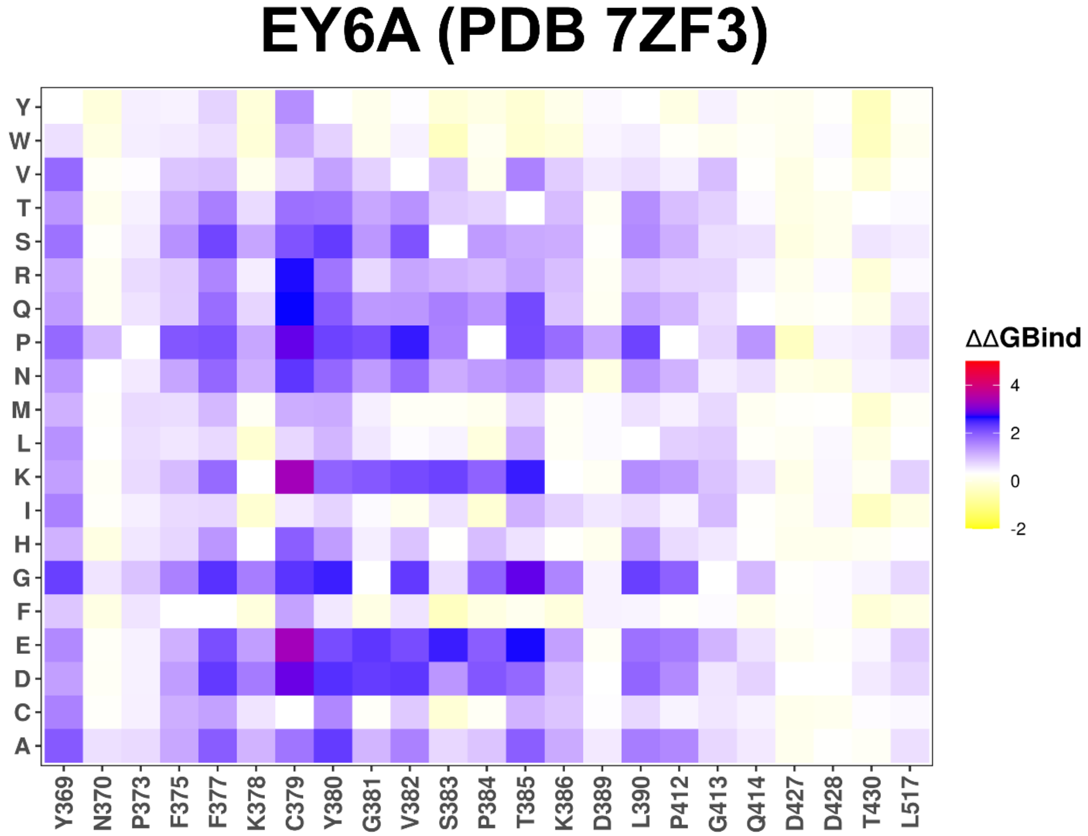
EY6A (PDB 7ZF3)
Y
W
V
T
S
R
Q
P
N
M
L
K
I
H
G
F
E
D
C
A
Y369
N370
P373
F375
F377
K378
C379
Y380
G381
V382
S383
P384
T385
K386
D389
L390
P412
G413
Q414
D427
D428
T430
L517
ΔΔGBind
4
2
0
-2

Figure S3. Mutational scanning of binding for the RBD complexes with SCORE-C epitope class CR3022 and EY6A antibodies. The mutational scanning heatmaps for the binding epitope residues in the S-RBD complexes with CR3022 (top panel) and EY6A (bottom panel). The heatmaps show the computed binding free energy changes for 20 single mutations on the sites of variants. The squares on the heatmap are colored using a 4-colored scale blue-white-yellow-red, with blue indicating the largest unfavorable effect on binding and stability, while yellow-red points to mutations that have favorable effect and improve binding.

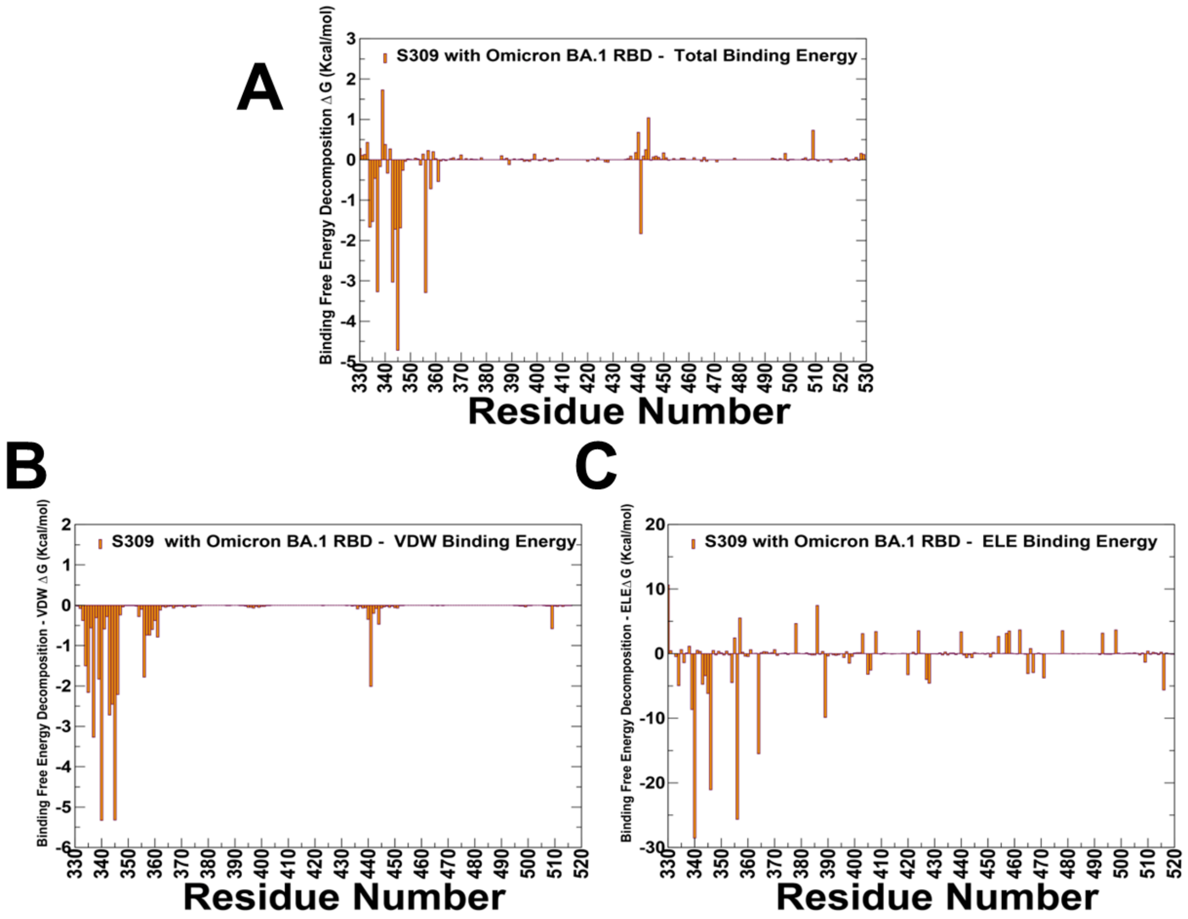

Figure S4. The residue-based decomposition of the binding MM-GBSA energies (A), van der Waals contributions (B) and electrostatic interactions (C) for the S-RBD complexes with SCORE-A S309 antibody. The binding free energy with MM-GBSA was computed by averaging the results of computations over 10,000 samples from the equilibrium ensembles. The standard error of the mean (SEM) for binding free energy estimates was calculated from the distribution of values obtained across the 10,000 snapshots sampled for each system. The statistical errors was estimated on the basis of the deviation between block average and are within 0.0.9 kcal/mol.

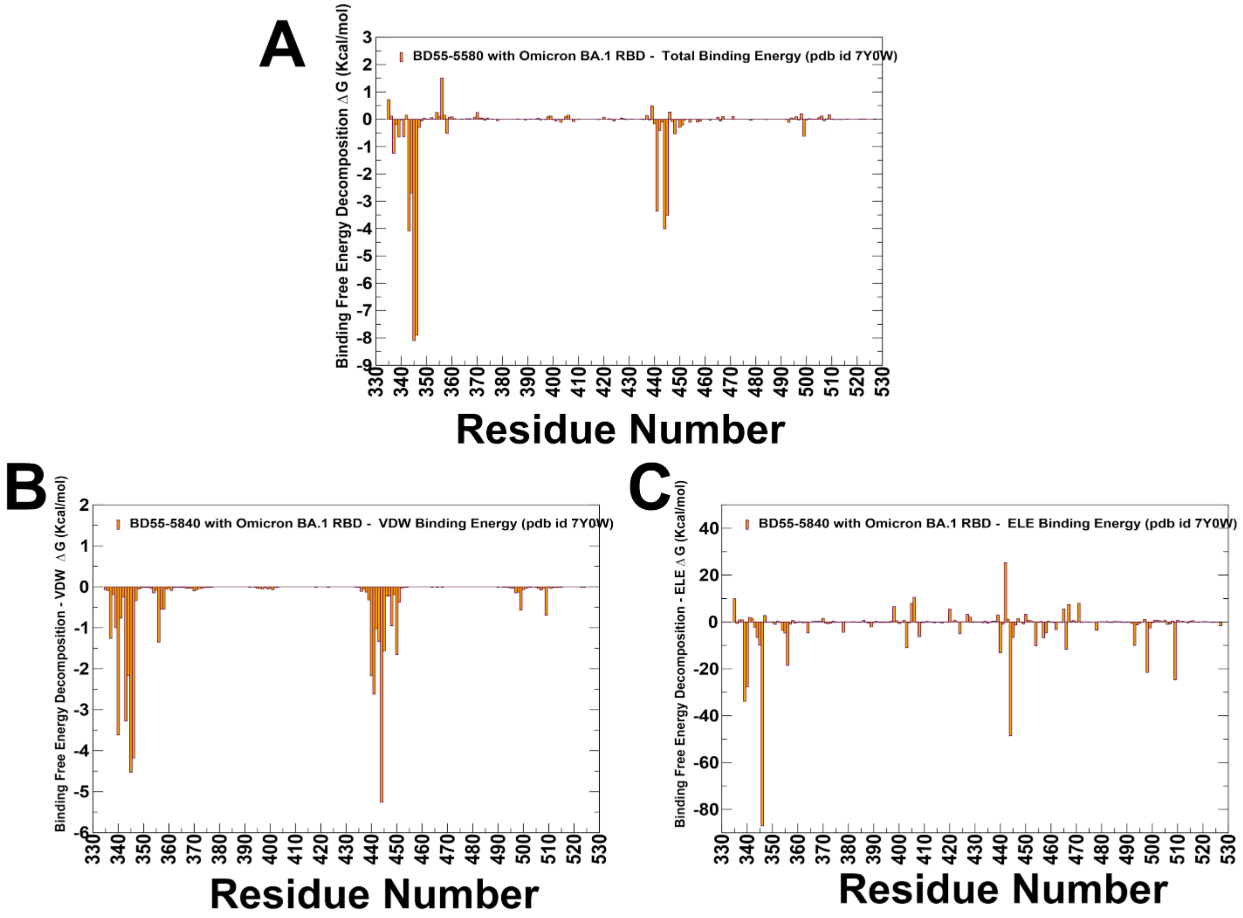

Figure S5. The residue-based decomposition of the binding MM-GBSA energies (A), van der Waals contributions (B) and electrostatic interactions (C) for the S-RBD complexes with SCORE-A SA58 antibody. The binding free energy with MM-GBSA was computed by averaging the results of computations over 10,000 samples from the equilibrium ensembles. The standard error of the mean (SEM) for binding free energy estimates was calculated from the distribution of values obtained across the 10,000 snapshots sampled for each system. The statistical errors was estimated on the basis of the deviation between block average and are within 0.15 kcal/mol.

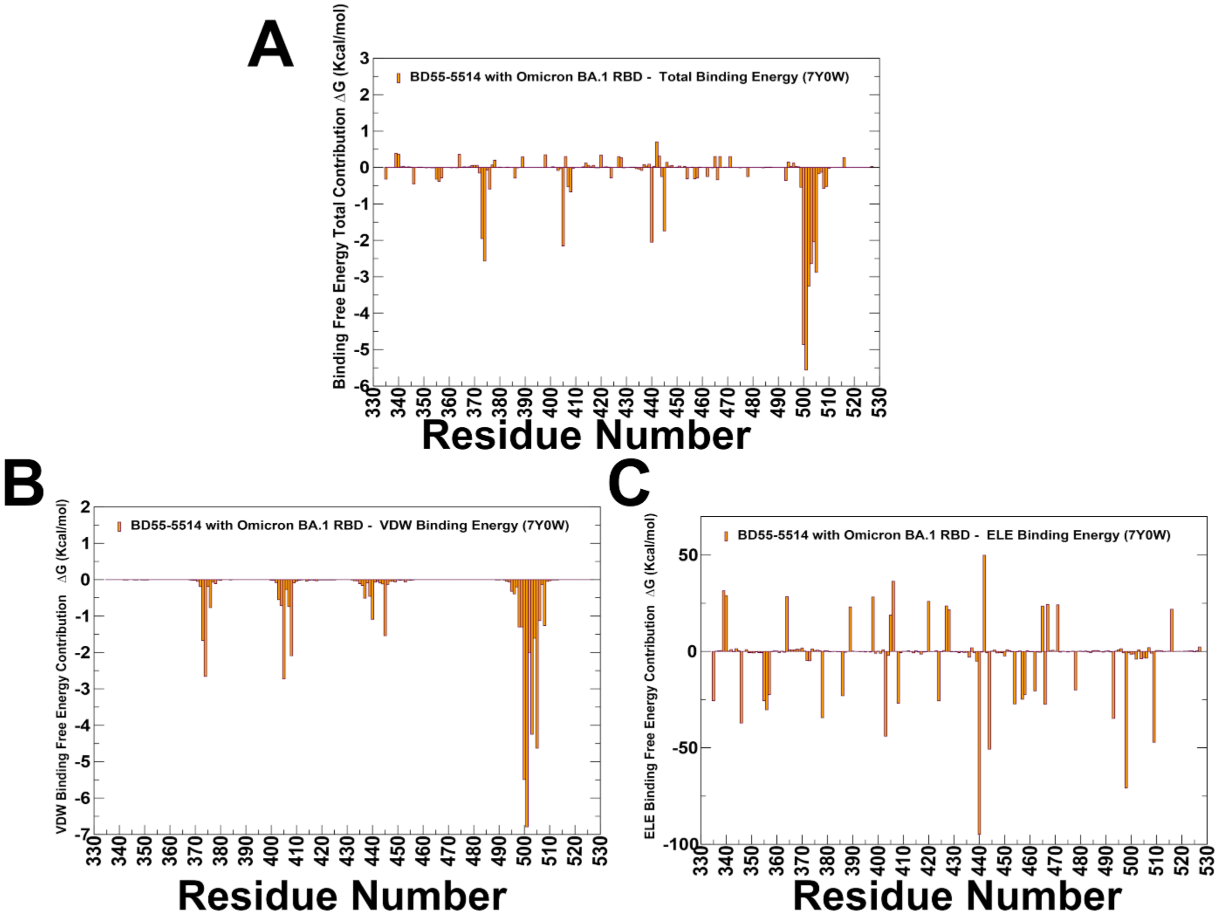

Figure S6. The residue-based decomposition of the binding MM-GBSA energies (A), van der Waals contributions (B) and electrostatic interactions (C) for the S-RBD complexes with SCORE-B SA55 antibody. The binding free energy with MM-GBSA was computed by averaging the results of computations over 10,000 samples from the equilibrium ensembles. The standard error of the mean (SEM) for binding free energy estimates was calculated from the distribution of values obtained across the 10,000 snapshots sampled for each system. The statistical errors was estimated on the basis of the deviation between block average and are within 0.22 kcal/mol.

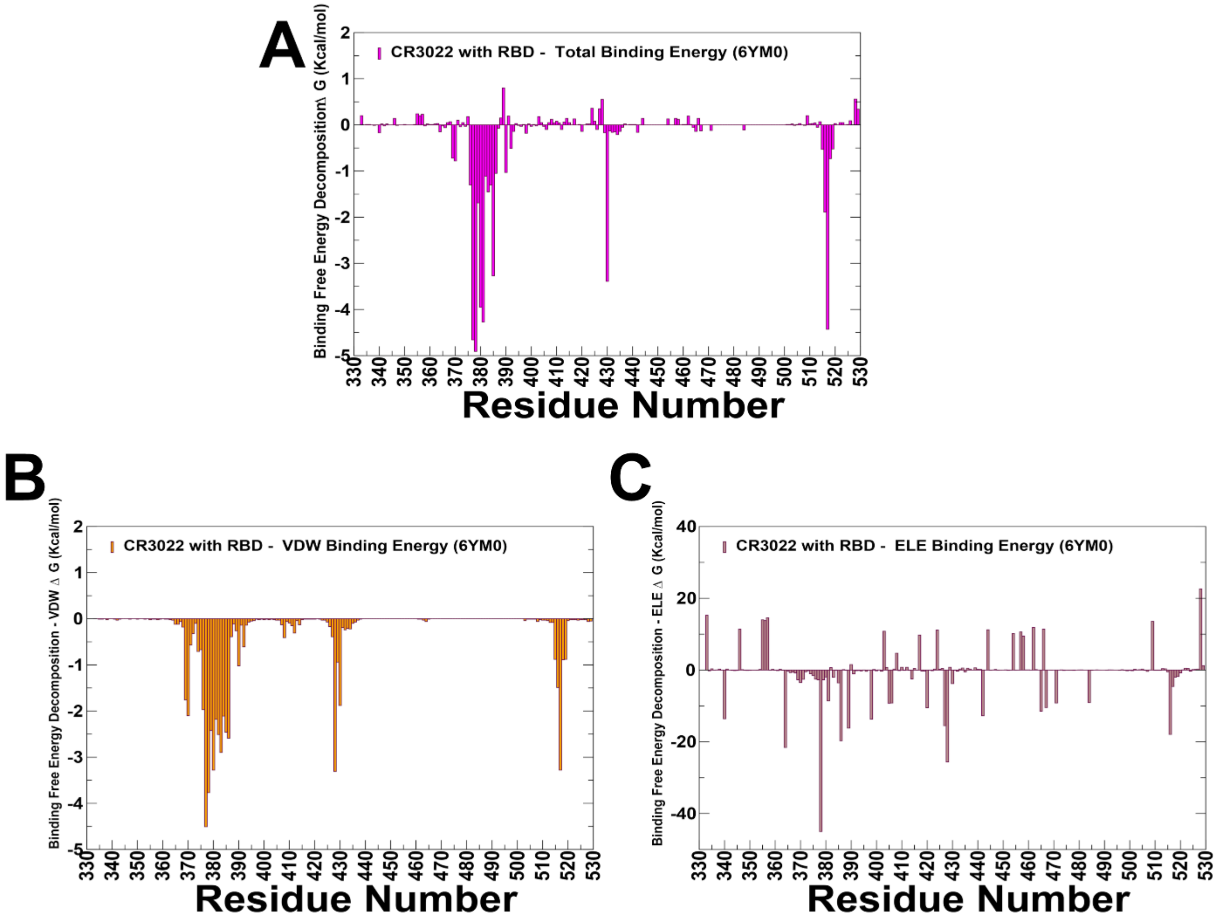

Figure S7. The residue-based decomposition of the binding MM-GBSA energies (A), van der Waals contributions (B) and electrostatic interactions (C) for the S-RBD complexes with SCORE-C CR3022 antibody. The binding free energy with MM-GBSA was computed by averaging the results of computations over 10,000 samples from the equilibrium ensembles. The standard error of the mean (SEM) for binding free energy estimates was calculated from the distribution of values obtained across the 10,000 snapshots sampled for each system. The statistical errors was estimated on the basis of the deviation between block average and are within 0.11 kcal/mol.

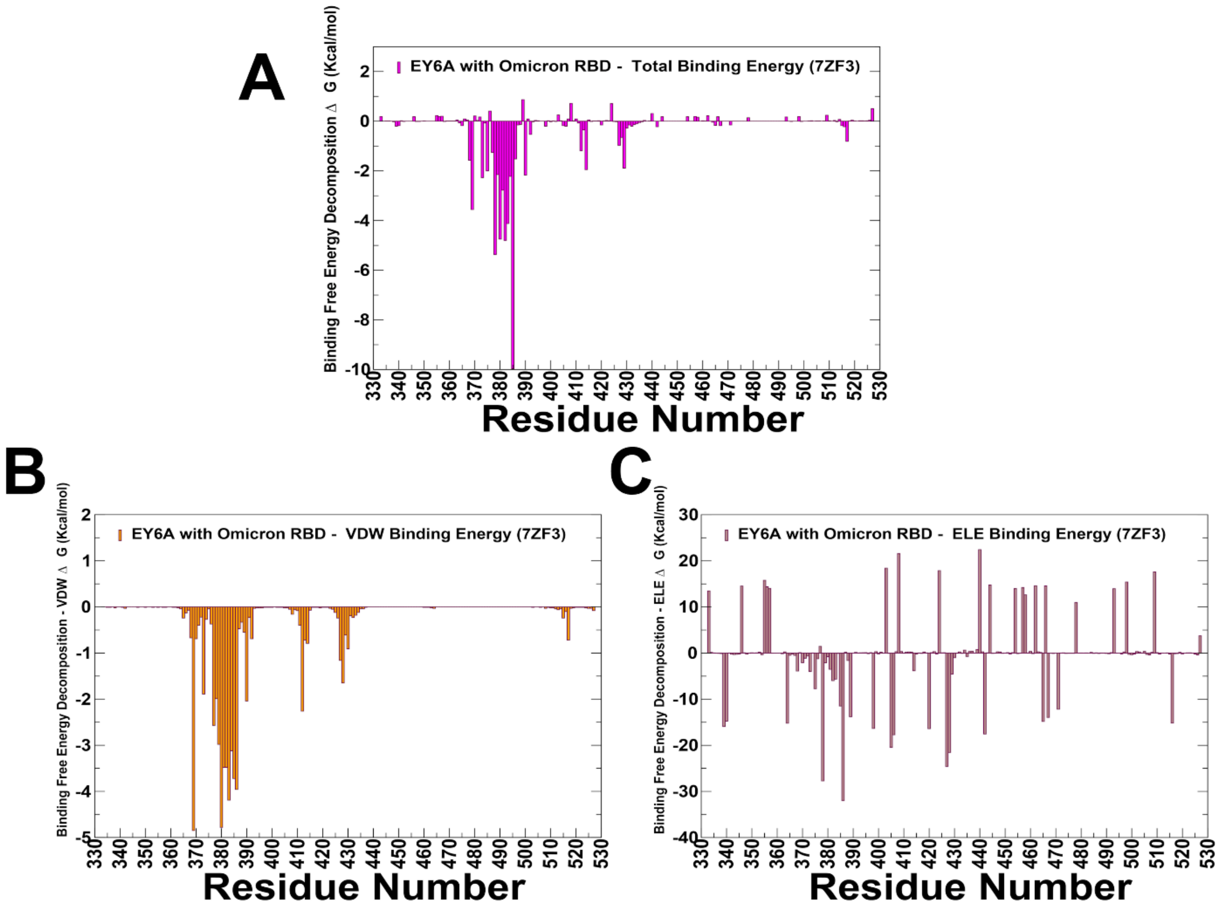

Figure S8. The residue-based decomposition of the binding MM-GBSA energies (A), van der Waals contributions (B) and electrostatic interactions (C) for the S-RBD complexes with SCORE-C EY6A antibody. The binding free energy with MM-GBSA was computed by averaging the results of computations over 10,000 samples from the equilibrium ensembles. The standard error of the mean (SEM) for binding free energy estimates was calculated from the distribution of values obtained across the 10,000 snapshots sampled for each system. The statistical errors was estimated on the basis of the deviation between block average and are within 0.15-0.17 kcal/mol.

Table S1. Major SARS-CoV-2 variants from Omicron to recent sublineages.

Variant / Sublineage	Lineage / Origin	Key RBD mutations (relative to previous)	Notable characteristics
**BA.1 (Omicron)**	Original Omicron	G339D, S373P, S375F, K417N, N440K, G446S, S477N, T478K, E484A, Q493R, G496S, Q498R, N501Y, Y505H	Major immune evasion; reduced ACE2 affinity vs. Delta
**BA.2**	BA.1 descendant	G339D, S373P, S375F, T376A, D405N, R408S, K417N, N440K, S477N, T478K, E484A, Q498R, N501Y, Y505H	More transmissible than BA.1; re-emergence of certain substitutions
**BA.4 / BA.5**	BA.2 descendants	Added L452R, F486V	Strong ACE2 affinity; significant escape from vaccine/ infection immunity
**XBB.1**	Recombination (BA.2 lineages)	G339H, R346T, L368Q, V445P, G446S, N460K, F486S, F490S	Growth advantage; moderate immune evasion
**XBB.1.5**	XBB.1 descendant	Added F486P	Enhanced ACE2 binding; became dominant globally
**BA.2.86**	BA.2 distant descendant	S50L, V127F, R158G, F186L, R190S, V213G, D339H, R346T, N354D, L452W, A484K, F486P	Highly mutated (over 30 S mutations); moderate ACE2 affinity
**JN.1**	BA.2.86 descendant	Added L455S (in RBD); other changes: S31del, F59S, Q183E, R190S, V213G, D339H, R346T, N354D, L452W, A484K, F486P	High immune evasion; became dominant worldwide
**KP.2**	JN.1 descendant	R346T, F456L, Q493E	Enhanced ACE2 binding (F456L + Q493E epistasis)
**KP.3**	JN.1 descendant	R346T, F456L, Q493E (similar to KP.2)	Among most antibody-evasive JN.1 sublineages
**XEC**	KP.3 recombinant	Additional: F59S, T22N (NTD)	Higher infectivity than KP.3; enhanced resistance

**LF.7**	JN.1 descendant	T22N, S31P, K182R, R190S (NTD) + R346T, K444R, F456L (RBD)	Multiple NTD + RBD mutations; transient prevalence
**LF.7.2.1**	LF.7 descendant	Added A475V	Outcompeted LF.7
**XFG ("Stratus")**	Recombinant (LF.7 × LP.8.1.2)	Additional: H445R, N487D, Q493E (RBD); T572I (SD1)	Became dominant global driver (as of March 2026)
**BA.3.2**	New lineage (distinct from JN.1)	20 RBD + 35 NTD differences vs. LP.8.1	High immune evasion coupled with attenuated replication fitness
**NB.1.8.1**	XDV recombinant	T22N, F59S, G184S (NTD) + A435S, F456L, K478I, Q493E (RBD)	Pronounced growth advantage
**KP.3.1.1**	KP.3 descendant	Deletion at S31 (NTD)	Enhanced ACE2 binding via F456L + Q493E epistasis
**LP.8.1 / LP.8.1.1**	JN.1 descendants	–	Displaced XEC in Europe/North America
**MC.10.1**	JN.1 descendant	–	Brief surge in global prevalence

Notes:

- This table focuses on variants/sublineages mentioned in the Introduction (lines 97–129).
- RBD mutations are listed for those with known impact on ACE2 binding or antibody escape.
- NTD = N-terminal domain; SD1 = subdomain 1.
- "–" indicates that specific RBD mutations are not highlighted in the main text for that variant.

For full mutation sets, please refer to original publications cited in the manuscript

Table S2. Classification systems for RBD-targeting SARS-CoV-2 neutralizing antibodies.

Classification	Epitope location / key feature	Neutralization potency	Escape susceptibility	Representative antibodies (including XGI)
**Barnes class 1**	"Up" RBD, overlaps ACE2-binding motif (RBS)	High	High (readily escaped by variants)	CB6/LY-CoV016, BD55-1205, VIR-7229, 19-77, ZCP3B4, ZCP4C9
**Barnes class 2**	"Down" RBD, also targets ACE2-binding region	High	High	–
**Barnes class 3**	Side of RBD, away from RBM	Moderate	Low to moderate	Corresponds to Cao group E; e.g., S2H97, COVOX-45, 553-49, XMA09
**Barnes class 4**	Cryptic / inner face (up conformation only)	Moderate to low	Very low (highly conserved epitope)	Cao group F (F1, F2, F3); **SCORE-A, SCORE-B, SCORE-C**; pemivibart (VYD222), SA55 (BD55-5514), **XGI antibodies**
**Cao groups A–D**	Subdivisions of Barnes classes 1/2 based on DMS escape patterns	High	Variable (group-dependent)	BD55-1205 (group A/B)
**Cao group E**	Corresponds to Barnes class 3 (side of RBD)	Moderate	Low	S2H97, COVOX-45, 553-49, XMA09
**Cao group F (F1/F2/F3)**	Corresponds to Barnes class 4 (inner face); F3 includes SCORE epitopes	Moderate (F3)	Very low	SA55, pemivibart, **XGI-183 (SCORE-A)**, **XGI-198/203 (SCORE-B)**, **XGI-171 (SCORE-C)**

**RBD-1/2**	Barnes class 1/2 (ACE2-overlapping)	High	High	–
**RBD-3/4/5**	Barnes class 3 (side of RBD)	Moderate	Low	–
**RBD-6/7**	Barnes class 4 (inner face)	Moderate	Very low	–
**SCORE-A**	Lateral RBD (α2-helix + β4-β5 hairpin), adjacent to NTD interface	Moderate	Moderate (K356T, R357T)	XGI-183, S309, SA58 (BD55-5840)
**SCORE-B**	RBM apex, overlaps ACE2 footprint	High (XGI-198) to moderate (XGI-203)	Low (conserved core) to moderate (periphery)	XGI-198, XGI-203, SA55
**SCORE-C**	Cryptic inner face, accessible only in "up" conformation	Low (allosteric loosening)	Very low (minimally frustrated core)	XGI-171, CR3022, EY6A

Table S3. The list of the intermolecular contacts in the structure of the XGI-183 antibody complex with RBD (pdb id 9KZE). The interfacial contacts in the structure are defined by counting the number of interatomic contacts within a 5.5 Å distance threshold between atoms of the interacting proteins.

RBD Residue	RBD Residue Number	RBD chain	Ab Residue	Ab Residue Number	Ab chain
PRO	337	A	GLY	29	L
PRO	337	A	ASN	80	L
GLU	340	A	GLY	29	L
GLU	340	A	SER	36	L
GLU	340	A	ASN	80	L
GLU	340	A	SER	83	L
GLU	340	A	GLY	84	L
VAL	341	A	GLY	29	L
VAL	341	A	SER	36	L
ASN	343	A	ASN	27	L
ALA	344	A	ASN	27	L
ALA	344	A	SER	36	L
THR	345	A	ASN	26	L
THR	345	A	ASN	27	L
THR	346	A	ASN	26	L
THR	346	A	ASN	27	L
THR	346	A	SER	109	L
PHE	347	A	SER	109	L
ALA	348	A	SER	109	L
SER	349	A	SER	109	L
TYR	351	A	TRP	107	L
TYR	351	A	SER	109	L
TYR	351	A	SER	110	L
TYR	351	A	PHE	113	L
TYR	351	A	ASP	114	L
ALA	352	A	TRP	107	L
ALA	352	A	ASP	108	L
ALA	352	A	SER	109	L
ALA	352	A	SER	110	L
ALA	352	A	ASP	114	L
TRP	353	A	LEU	110	H
TRP	353	A	TRP	107	L
ASN	354	A	SER	36	L
ASN	354	A	LYS	37	L
ASN	354	A	TRP	107	L
ASN	354	A	ASP	108	L

ASN	354	A	SER	109	L
ARG	355	A	LEU	110	H
ARG	355	A	THR	112	H
ARG	355	A	ASN	38	L
ARG	355	A	TRP	107	L
LYS	356	A	ILE	28	L
LYS	356	A	GLY	29	L
LYS	356	A	SER	36	L
LYS	356	A	LYS	37	L
LYS	356	A	ASN	38	L
LYS	356	A	VAL	39	L
LYS	356	A	ASP	57	L
LYS	356	A	ASN	80	L
ARG	357	A	THR	112	H
ARG	357	A	ASN	38	L
ARG	357	A	TYR	55	L
ARG	357	A	ASP	56	L
ARG	357	A	SER	65	L
ARG	357	A	ASP	66	L
ILE	358	A	ASN	38	L
SER	359	A	ASP	56	L
SER	359	A	SER	65	L
SER	359	A	ASP	66	L
ASN	360	A	SER	65	L
ASN	360	A	ASP	66	L
ARG	457	A	SER	63	H
ARG	457	A	ASN	64	H
LEU	461	A	ASN	64	H
LYS	462	A	TYR	58	H
PHE	464	A	LEU	110	H
GLU	465	A	SER	57	H
GLU	465	A	TYR	58	H
GLU	465	A	ASN	64	H
GLU	465	A	LEU	110	H
ARG	466	A	ASN	64	H
ARG	466	A	GLU	107	H
ARG	466	A	LEU	110	H
ARG	466	A	THR	112	H
ARG	466	A	TRP	107	L
ARG	466	A	TRP	116	L
ASP	467	A	ASN	64	H
ILE	468	A	VAL	55	H
ILE	468	A	ASN	64	H

ILE	468	A	LYS	65	H
ILE	468	A	HIS	66	H
ILE	468	A	TRP	107	L
ILE	468	A	PHE	113	L
ILE	468	A	ASP	114	L
ILE	468	A	TRP	116	L
SER	469	A	SER	63	H
SER	469	A	ASN	64	H
SER	469	A	LYS	65	H
SER	469	A	HIS	66	H
THR	470	A	LYS	65	H
THR	470	A	HIS	66	H
THR	470	A	LYS	72	H
THR	470	A	PHE	113	L
GLU	471	A	LYS	65	H
GLU	471	A	HIS	66	H
GLU	471	A	TYR	67	H
GLU	471	A	LYS	72	H
LEU	492	A	PHE	113	L

Table S4. The list of the intermolecular contacts in the structure of the S309 antibody complex with RBD (pdb id 7YAD). The interfacial contacts in the structure are defined by counting the number of interatomic contacts within a 5.5 Å distance threshold between atoms of the interacting proteins.

RBD Residue	RBD Residue Number	RBD chain	Ab Residue	Ab Residue Number	Ab chain
ASN	334	M	TYR	54	A
ASN	334	M	THR	30	A
ASN	334	M	TRP	105	A
LEU	335	M	SER	31	A
LEU	335	M	PRO	28	A
LEU	335	M	TRP	105	A
CYS	336	M	TRP	105	A
CYS	336	M	SER	31	A
PRO	337	M	PHE	106	A
PRO	337	M	TRP	105	A
ASP	339	M	TYR	32	A
ASP	339	M	LEU	110	A
ASP	339	M	TYR	100	A
ASP	339	M	SER	31	A
GLU	340	M	GLY	107	A
GLU	340	M	TRP	105	A
GLU	340	M	ARG	102	A
GLU	340	M	GLY	103	A
GLU	340	M	SER	31	A
GLU	340	M	PHE	106	A
GLU	340	M	ALA	104	A
GLU	340	M	GLU	108	A
GLU	340	M	LEU	110	A
VAL	341	M	LEU	110	A
VAL	341	M	PHE	106	A
ASN	343	M	TYR	100	A
ASN	343	M	LEU	110	A
ASN	343	M	ILE	111	A
ASN	343	M	SER	109	A
ALA	344	M	SER	109	A
ALA	344	M	LEU	110	A
ALA	344	M	GLU	108	A
ALA	344	M	ILE	111	A
THR	345	M	LEU	110	A
THR	345	M	THR	32	B
THR	345	M	ILE	111	A

THR	345	M	SER	33	B
THR	345	M	HIS	92	B
THR	345	M	SER	109	A
ARG	346	M	GLU	108	A
ARG	346	M	SER	109	A
ARG	346	M	SER	30	B
ARG	346	M	ASP	93	B
ASN	354	M	GLU	108	A
LYS	356	M	PHE	106	A
LYS	356	M	GLU	108	A
ARG	357	M	PHE	106	A
ILE	358	M	TRP	105	A
ILE	358	M	PHE	106	A
SER	359	M	TRP	105	A
SER	359	M	TYR	54	A
ASN	360	M	TRP	105	A
CYS	361	M	TRP	105	A
LYS	440	M	SER	31	B
LEU	441	M	SER	31	B
LEU	441	M	THR	32	B
LEU	441	M	ILE	111	A
ARG	509	M	THR	32	B
ARG	509	M	ILE	111	A

Table S5. The list of the intermolecular contacts in the structure of the SA58 antibody complex with RBD (pdb id 7Y0W). The interfacial contacts in the structure are defined by counting the number of interatomic contacts within a 5.5 Å distance threshold between atoms of the interacting proteins.

RBD Residue	RBD Residue Number	RBD chain	Ab Residue	Ab Residue Number	Ab chain
PRO	337	R	LEU	29	L
PRO	337	R	SER	28	L
ASP	339	R	ASN	95	L
ASP	339	R	GLU	1	L
GLU	340	R	ALA	27	L
GLU	340	R	SER	28	L
GLU	340	R	ASN	95	L
GLU	340	R	GLU	1	L
GLU	340	R	ARG	26	L
GLU	340	R	GLY	30	L
GLU	340	R	LEU	29	L
GLU	340	R	VAL	2	L
VAL	341	R	ASN	95	L
VAL	341	R	LEU	29	L
ASN	343	R	SER	94	L
ASN	343	R	PRO	97	L
ASN	343	R	ASN	95	L
ASN	343	R	TRP	96	L
ALA	344	R	SER	94	L
ALA	344	R	ASN	95	L
ALA	344	R	TRP	96	L
THR	345	R	TRP	96	L
THR	345	R	TYR	105	H
THR	345	R	ASP	34	L
THR	345	R	TYR	93	L
THR	345	R	LEU	98	L
THR	345	R	SER	94	L
THR	345	R	ASN	95	L
ARG	346	R	PHE	106	H
ARG	346	R	SER	94	L
ARG	346	R	SER	103	H
ARG	346	R	ASP	104	H
ARG	346	R	TYR	105	H
ARG	346	R	ASP	34	L
ARG	346	R	TYR	93	L
LYS	356	R	LEU	29	L

ARG	357	R	LEU	29	L
ILE	358	R	LEU	29	L
LYS	440	R	TRP	50	H
LYS	440	R	THR	57	H
LYS	440	R	ASN	32	H
LYS	440	R	PRO	58	H
LYS	440	R	ASN	52	H
LYS	440	R	TYR	102	H
LYS	440	R	THR	59	H
LEU	441	R	ASN	52	H
LEU	441	R	TRP	96	L
LEU	441	R	TYR	102	H
LEU	441	R	TRP	50	H
LEU	441	R	SER	103	H
LEU	441	R	TYR	105	H
ASP	442	R	TYR	102	H
ASP	442	R	SER	103	H
ASP	442	R	TYR	105	H
SER	443	R	ASP	54	H
SER	443	R	TYR	102	H
SER	443	R	ASN	32	H
LYS	444	R	THR	30	H
LYS	444	R	ASN	32	H
LYS	444	R	SER	31	H
LYS	444	R	ASP	54	H
LYS	444	R	TYR	102	H
VAL	445	R	ASP	54	H
ASN	448	R	TYR	102	H
ASN	448	R	SER	103	H
ASN	450	R	TYR	102	H
ASN	450	R	SER	103	H
TYR	451	R	SER	103	H
ARG	509	R	TYR	105	H
ARG	509	R	TRP	96	L

Table S6. The list of the intermolecular contacts in the structure of the XGI-188 antibody complex with RBD (pdb id 9L05). The interfacial contacts in the structure are defined by counting the number of interatomic contacts within a 5.5 Å distance threshold between atoms of the interacting proteins.

RBD Residue	RBD Residue Number	RBD chain	Ab Residue	Ab Residue Number	Ab chain
PRO	373	A	SER	59	H
PHE	375	A	SER	59	H
ASN	437	A	HIS	57	H
ASN	437	A	SER	59	H
ASN	437	A	ASN	64	H
SER	438	A	ASN	64	H
ASN	439	A	TYR	38	H
ASN	439	A	ASN	64	H
ASN	439	A	TYR	66	H
ASN	439	A	ASN	114	L
LYS	440	A	ASN	64	H
LYS	440	A	THR	65	H
LYS	440	A	TYR	66	H
LYS	444	A	ASN	109	L
PRO	445	A	ASN	109	L
PRO	445	A	SER	113	L
ARG	498	A	LEU	112	H
ARG	498	A	ASP	36	L
ARG	498	A	ASN	37	L
ARG	498	A	ASN	109	L
PRO	499	A	TYR	38	H
PRO	499	A	TYR	66	H
PRO	499	A	VAL	112	H
PRO	499	A	ASN	109	L
PRO	499	A	SER	113	L
PRO	499	A	ASN	114	L
THR	500	A	VAL	109	H
THR	500	A	VAL	112	H
THR	500	A	LEU	112	H
THR	500	A	ASP	36	L
THR	500	A	ASN	37	L
THR	500	A	TYR	107	L
THR	500	A	ASP	108	L
THR	500	A	ASN	109	L
THR	500	A	SER	113	L
THR	500	A	ASN	114	L

TYR	501	A	TYR	38	H
TYR	501	A	VAL	109	H
TYR	501	A	VAL	112	H
TYR	501	A	LEU	112	H
GLY	502	A	VAL	109	H
GLY	502	A	GLY	110	H
GLY	502	A	GLY	111	H
GLY	502	A	VAL	112	H
GLY	502	A	LEU	112	H
VAL	503	A	GLU	35	H
VAL	503	A	TYR	36	H
VAL	503	A	TYR	38	H
VAL	503	A	HIS	57	H
VAL	503	A	VAL	109	H
VAL	503	A	GLY	110	H
GLY	504	A	TYR	36	H
GLY	504	A	VAL	109	H
GLN	506	A	TYR	38	H
GLN	506	A	ASN	64	H
GLN	506	A	TYR	66	H
GLN	506	A	VAL	109	H
TYR	508	A	HIS	57	H
TYR	508	A	SER	59	H

Table S7. The list of the intermolecular contacts in the structure of the XGI-203 antibody complex with RBD (pdb id 9L07). The interfacial contacts in the structure are defined by counting the number of interatomic contacts within a 5.5 Å distance threshold between atoms of the interacting proteins.

RBD Residue	RBD Residue Number	RBD chain	Ab Residue	Ab Residue Number	Ab chain
ALA	372	A	SER	59	H
ALA	372	A	GLY	63	H
PRO	373	A	TYR	58	H
PRO	373	A	SER	59	H
PRO	373	A	GLY	63	H
PHE	374	A	TYR	58	H
PHE	375	A	ASN	34	H
PHE	375	A	TYR	37	H
PHE	375	A	TYR	58	H
ASN	437	A	HIS	57	H
ASN	437	A	SER	59	H
ASN	437	A	ASN	64	H
ASN	437	A	TYR	66	H
SER	438	A	ASN	64	H
ASN	439	A	TYR	38	H
ASN	439	A	ASN	64	H
ASN	439	A	TYR	66	H
ASN	439	A	ASN	114	L
LYS	440	A	GLY	63	H
LYS	440	A	ASN	64	H
LYS	440	A	THR	65	H
LYS	440	A	TYR	66	H
LYS	444	A	ASN	109	L
PRO	445	A	ASN	109	L
PRO	445	A	SER	113	L
SER	446	A	ASN	109	L
ARG	498	A	LEU	112	H
ARG	498	A	ASP	36	L
ARG	498	A	ASN	109	L
PRO	499	A	TYR	38	H
PRO	499	A	TYR	66	H
PRO	499	A	VAL	112	H
PRO	499	A	ASN	109	L
PRO	499	A	SER	113	L
PRO	499	A	ASN	114	L
THR	500	A	VAL	112	H

THR	500	A	LEU	112	H
THR	500	A	ASP	36	L
THR	500	A	ASN	37	L
THR	500	A	TYR	107	L
THR	500	A	ASP	108	L
THR	500	A	ASN	109	L
THR	500	A	SER	113	L
THR	500	A	ASN	114	L
THR	500	A	VAL	115	L
TYR	501	A	VAL	109	H
TYR	501	A	GLY	111	H
TYR	501	A	VAL	112	H
TYR	501	A	LEU	112	H
GLY	502	A	VAL	109	H
GLY	502	A	GLY	110	H
GLY	502	A	GLY	111	H
GLY	502	A	VAL	112	H
GLY	502	A	LEU	112	H
VAL	503	A	TYR	37	H
VAL	503	A	HIS	57	H
VAL	503	A	VAL	109	H
VAL	503	A	GLY	110	H
VAL	503	A	GLY	111	H
GLY	504	A	VAL	109	H
GLY	504	A	GLY	111	H
HIS	505	A	GLY	111	H
GLN	506	A	TYR	38	H
GLN	506	A	HIS	57	H
GLN	506	A	ASN	64	H
GLN	506	A	TYR	66	H
GLN	506	A	VAL	109	H
GLN	506	A	VAL	112	H
GLN	506	A	ASN	114	L
TYR	508	A	HIS	57	H

Table S8. The list of the intermolecular contacts in the structure of the SA55 antibody complex with RBD (pdb id 7Y0W). The interfacial contacts in the structure are defined by counting the number of interatomic contacts within a 5.5 Å distance threshold between atoms of the interacting proteins.

RBD Residue	RBD Residue Number	RBD chain	Ab Residue	Ab Residue Number	Ab chain
PRO	373	R	LEU	94	B
PHE	374	R	THR	57	A
PHE	374	R	PHE	55	A
THR	376	R	PHE	55	A
ARG	403	R	PRO	105	A
ARG	403	R	ASN	106	A
GLY	404	R	PHE	55	A
GLY	404	R	LEU	54	A
GLY	404	R	ARG	30	A
ASP	405	R	LEU	54	A
ASP	405	R	SER	31	A
ASP	405	R	THR	28	A
ASP	405	R	ARG	30	A
GLU	406	R	ARG	30	A
VAL	407	R	ARG	30	A
VAL	407	R	PHE	55	A
VAL	407	R	LEU	54	A
ARG	408	R	ARG	30	A
ASN	437	R	ASP	93	B
ASN	439	R	TYR	91	B
ASN	439	R	ASP	93	B
LYS	440	R	ASP	93	B
VAL	445	R	HIS	53	B
TYR	495	R	PRO	105	A
SER	496	R	PRO	105	A
ARG	498	R	PHE	112	A
ARG	498	R	TYR	49	B
PRO	499	R	PHE	100	A
PRO	499	R	PRO	101	A
PRO	499	R	ASP	50	B
PRO	499	R	TYR	91	B
THR	500	R	GLY	103	A
THR	500	R	PHE	112	A
THR	500	R	PHE	100	A
THR	500	R	TYR	49	B
THR	500	R	ASP	104	A

THR	500	R	PRO	101	A
THR	500	R	ASN	102	A
THR	500	R	ASP	50	B
TYR	501	R	PRO	105	A
TYR	501	R	GLY	103	A
TYR	501	R	PHE	112	A
TYR	501	R	ASN	102	A
TYR	501	R	ASP	104	A
TYR	501	R	PRO	101	A
GLY	502	R	ASP	104	A
GLY	502	R	PRO	101	A
GLY	502	R	SER	31	A
GLY	502	R	GLY	103	A
GLY	502	R	HIS	32	A
GLY	502	R	ASN	102	A
VAL	503	R	PRO	95	B
VAL	503	R	PHE	55	A
VAL	503	R	VAL	33	A
VAL	503	R	ASN	102	A
VAL	503	R	LEU	54	A
VAL	503	R	HIS	32	A
VAL	503	R	PRO	101	A
VAL	503	R	ILE	52	A
VAL	503	R	SER	31	A
GLY	504	R	LEU	54	A
GLY	504	R	HIS	32	A
GLY	504	R	SER	31	A
GLY	504	R	ARG	30	A
HIS	505	R	PRO	105	A
HIS	505	R	HIS	32	A
HIS	505	R	ASP	104	A
HIS	505	R	SER	31	A
HIS	505	R	GLY	103	A
GLN	506	R	PRO	101	A
GLN	506	R	TYR	91	B
GLN	506	R	ASP	93	B
TYR	508	R	LEU	54	A
TYR	508	R	PHE	55	A

Table S9. The list of the intermolecular contacts in the structure of the XGI-171 antibody complex with RBD (pdb id 9KZD). The interfacial contacts in the structure are defined by counting the number of interatomic contacts within a 5.5 Å distance threshold between atoms of the interacting proteins.

RBD Residue	RBD Residue Number	RBD chain	Ab Residue	Ab Residue Number	Ab chain
TYR	369	A	ASP	64	H
TYR	369	A	TYR	66	H
PRO	373	A	LYS	72	H
PHE	374	A	LYS	72	H
PHE	375	A	LYS	72	H
PHE	377	A	TYR	66	H
PHE	377	A	PRO	114	L
LYS	378	A	ASP	1	L
LYS	378	A	SER	109	L
LYS	378	A	PRO	114	L
LYS	378	A	PRO	115	L
CYS	379	A	TYR	66	H
CYS	379	A	TYR	108	L
CYS	379	A	SER	109	L
CYS	379	A	MET	113	L
CYS	379	A	PRO	114	L
CYS	379	A	PRO	115	L
TYR	380	A	ILE	2	L
TYR	380	A	TYR	108	L
TYR	380	A	SER	109	L
TYR	380	A	MET	113	L
TYR	380	A	PRO	114	L
GLY	381	A	GLN	111	H
GLY	381	A	PHE	38	L
GLY	381	A	SER	107	L
GLY	381	A	TYR	108	L
GLY	381	A	SER	109	L
GLY	381	A	MET	113	L
VAL	382	A	GLN	111	H
VAL	382	A	TYR	108	L
VAL	382	A	MET	113	L
SER	383	A	GLY	110	H
SER	383	A	GLN	111	H
SER	383	A	PHE	112	H
SER	383	A	TRP	113	H
SER	383	A	MET	113	L

PRO	384	A	ASP	64	H
PRO	384	A	TYR	66	H
PRO	384	A	TRP	113	H
PRO	384	A	MET	113	L
PRO	384	A	PRO	114	L
THR	385	A	ASP	38	H
THR	385	A	ILE	56	H
THR	385	A	GLY	57	H
THR	385	A	THR	58	H
THR	385	A	ASP	64	H
THR	385	A	TYR	66	H
THR	385	A	TRP	113	H
LYS	386	A	ASP	38	H
LYS	386	A	GLY	108	H
LYS	386	A	SER	109	H
LYS	386	A	GLY	110	H
LYS	386	A	GLN	111	H
ASN	388	A	GLY	59	H
LEU	390	A	GLN	111	H
ALA	411	A	GLN	27	L
PRO	412	A	GLN	27	L
PRO	412	A	THR	28	L
PRO	412	A	TYR	108	L
GLY	413	A	GLN	27	L
PRO	426	A	TYR	108	L
ASP	427	A	THR	28	L
ASP	427	A	TYR	108	L
ASP	428	A	PHE	38	L
ASP	428	A	TYR	108	L
PHE	429	A	TYR	108	L
THR	430	A	TYR	108	L

Table S10. The list of the intermolecular contacts in the structure of the CR-3022 antibody complex with RBD (pdb id 6YM0). The interfacial contacts in the structure are defined by counting the number of interatomic contacts within a 5.5 Å distance threshold between atoms of the interacting proteins.

RBD Residue	RBD Residue Number	RBD chain	Ab Residue	Ab Residue Number	Ab chain
TYR	369	E	ILE	30	H
TYR	369	E	THR	31	H
TYR	369	E	GLY	28	H
TYR	369	E	PHE	29	H
TYR	369	E	TYR	27	H
ASN	370	E	GLY	28	H
ASN	370	E	TYR	27	H
SER	371	E	ILE	30	H
PHE	374	E	ILE	30	H
SER	375	E	TYR	52	H
SER	375	E	ILE	30	H
SER	375	E	GLY	54	H
THR	376	E	ILE	30	H
THR	376	E	TYR	52	H
THR	376	E	GLY	54	H
THR	376	E	ASP	55	H
PHE	377	E	THR	31	H
PHE	377	E	TRP	33	H
PHE	377	E	TYR	52	H
PHE	377	E	TYR	32	H
PHE	377	E	ILE	30	H
LYS	378	E	ILE	30	H
LYS	378	E	THR	31	H
LYS	378	E	TRP	33	H
LYS	378	E	ASP	55	H
LYS	378	E	GLU	57	H
LYS	378	E	TYR	52	H
CYS	379	E	THR	31	H
CYS	379	E	TRP	33	H
CYS	379	E	ILE	102	H
CYS	379	E	SER	100	H
CYS	379	E	GLY	101	H
TYR	380	E	TRP	33	H
TYR	380	E	ARG	59	H
TYR	380	E	THR	104	H
TYR	380	E	ILE	102	H

TYR	380	E	GLU	57	H
TYR	380	E	GLY	101	H
TYR	380	E	SER	103	H
GLY	381	E	ILE	34	L
GLY	381	E	TYR	31	L
GLY	381	E	THR	104	H
GLY	381	E	TYR	38	L
GLY	381	E	GLY	101	H
GLY	381	E	ILE	102	H
GLY	381	E	SER	103	H
GLY	381	E	TRP	56	L
VAL	382	E	GLY	101	H
VAL	382	E	ILE	102	H
VAL	382	E	SER	103	H
VAL	382	E	SER	100	H
VAL	382	E	TRP	56	L
VAL	382	E	ILE	34	L
VAL	382	E	THR	104	H
VAL	382	E	TYR	38	L
SER	383	E	GLY	101	H
SER	383	E	SER	100	H
SER	383	E	GLY	99	H
SER	383	E	THR	104	H
SER	383	E	PRO	105	H
PRO	384	E	SER	100	H
PRO	384	E	THR	31	H
PRO	384	E	GLY	101	H
THR	385	E	TYR	32	H
THR	385	E	THR	31	H
THR	385	E	SER	100	H
THR	385	E	GLN	1	H
THR	385	E	ASP	107	H
LYS	386	E	PRO	105	H
LYS	386	E	TYR	55	L
LYS	386	E	GLU	61	L
LYS	386	E	LEU	52	L
LYS	386	E	ASP	107	H
LYS	386	E	SER	100	H
ASP	389	E	TYR	55	L
LEU	390	E	TRP	56	L
PHE	392	E	TRP	56	L
PHE	392	E	ILE	34	L
ARG	408	E	ASP	55	H

ASP	427	E	TYR	31	L
ASP	428	E	SER	32	L
ASP	428	E	TYR	31	L
ASP	428	E	SER	33	L
ASP	428	E	TYR	98	L
PHE	429	E	TYR	31	L
THR	430	E	ILE	34	L
THR	430	E	TYR	31	L
THR	430	E	SER	33	L
THR	430	E	TYR	38	L
PHE	515	E	SER	33	L
PHE	515	E	ILE	34	L
GLU	516	E	ILE	34	L
GLU	516	E	SER	33	L
LEU	517	E	ILE	34	L
LEU	517	E	ASN	35	L
LEU	517	E	SER	32	L
LEU	517	E	SER	33	L
LEU	517	E	LYS	36	L
LEU	518	E	SER	33	L
HIS	519	E	ASN	35	L

Table S11. The list of the intermolecular contacts in the structure of the EY6A antibody complex with RBD (pdb id 7ZF3). The interfacial contacts in the structure are defined by counting the number of interatomic contacts within a 5.5 Å distance threshold between atoms of the interacting proteins.

RBD Residue	RBD Residue Number	RBD chain	Ab Residue	Ab Residue Number	Ab chain
LEU	368	E	TYR	59	H
LEU	368	E	ASN	57	H
TYR	369	E	ASN	57	H
TYR	369	E	LYS	58	H
TYR	369	E	SER	56	H
TYR	369	E	TYR	59	H
ASN	370	E	LYS	58	H
ASN	370	E	SER	56	H
ASN	370	E	TYR	59	H
ASN	370	E	ASN	57	H
ALA	372	E	LYS	65	H
PRO	373	E	GLY	66	H
PRO	373	E	LYS	65	H
PHE	375	E	LYS	65	H
THR	376	E	LYS	65	H
PHE	377	E	TYR	59	H
PHE	377	E	LYS	65	H
PHE	377	E	LEU	95	L
LYS	378	E	LEU	95	L
LYS	378	E	SER	93	L
LYS	378	E	ALA	96	L
LYS	378	E	ASP	1	L
LYS	378	E	ASP	62	H
CYS	379	E	LEU	95	L
CYS	379	E	SER	93	L
CYS	379	E	TYR	92	L
CYS	379	E	THR	94	L
TYR	380	E	SER	93	L
TYR	380	E	TYR	92	L
TYR	380	E	THR	94	L
GLY	381	E	SER	93	L
GLY	381	E	TYR	92	L
GLY	381	E	THR	94	L
GLY	381	E	TRP	104	H
GLY	381	E	SER	91	L
GLY	381	E	TYR	32	L

GLY	381	E	VAL	105	H
VAL	382	E	TRP	104	H
VAL	382	E	TYR	32	L
VAL	382	E	VAL	105	H
VAL	382	E	TYR	92	L
VAL	382	E	THR	94	L
SER	383	E	TRP	104	H
SER	383	E	VAL	105	H
SER	383	E	THR	94	L
SER	383	E	TYR	106	H
PRO	384	E	TYR	59	H
PRO	384	E	THR	94	L
PRO	384	E	TYR	106	H
PRO	384	E	LEU	95	L
PRO	384	E	ASN	57	H
THR	385	E	VAL	50	H
THR	385	E	TYR	53	H
THR	385	E	TYR	106	H
THR	385	E	SER	52	H
THR	385	E	TYR	59	H
THR	385	E	ASP	33	H
THR	385	E	ASN	57	H
THR	385	E	ILE	51	H
LYS	386	E	TRP	104	H
LYS	386	E	ASP	33	H
LYS	386	E	GLY	101	H
LYS	386	E	VAL	105	H
LYS	386	E	LEU	103	H
LYS	386	E	ASP	99	H
LYS	386	E	TYR	106	H
LYS	386	E	LYS	102	H
ASN	388	E	TYR	53	H
ASP	389	E	TYR	53	H
LEU	390	E	TRP	104	H
PHE	392	E	TRP	104	H
ALA	411	E	GLN	27	L
PRO	412	E	GLN	27	L
PRO	412	E	TYR	92	L
GLY	413	E	GLN	27	L
GLN	414	E	GLN	27	L
PRO	426	E	TYR	92	L
ASP	427	E	SER	28	L
ASP	427	E	SER	30	L

ASP	427	E	TYR	92	L
ASP	428	E	SER	30	L
ASP	428	E	TYR	92	L
PHE	429	E	TYR	92	L
THR	430	E	TYR	92	L
THR	430	E	TRP	104	H
LEU	517	E	TRP	104	H

Table S12. Comparison of key observables between single trajectory and three independent replicates for XGI antibody-RBD complexes. Values are reported as mean ± SEM (standard error of the mean). Replicate averages are calculated from three independent 500 ns simulations. p-values are from paired two-tailed t-tests.

Complex	Observable	Single trajectory	Replicate average (n=3)	p-value
**XGI-171**	RMSF (Å) over RBD 330-530	0.92 ± 0.04	0.94 ± 0.03	0.31
	Number of inter-protein H-bonds	12.3 ± 1.1	12.1 ± 0.9	0.67
	Crystal contacts retained (%)	88 ± 3	86 ± 4	0.44
	Complex RMSD after 200 ns (Å)	2.1 ± 0.2	2.0 ± 0.2	0.51
**XGI-183**	RMSF (Å) over RBD 330-530	0.88 ± 0.05	0.86 ± 0.04	0.42
	Number of inter-protein H-bonds	14.7 ± 1.3	14.9 ± 1.2	0.58
	Crystal contacts retained (%)	86 ± 4	87 ± 3	0.61
	Complex RMSD after 200 ns (Å)	1.9 ± 0.2	2.0 ± 0.2	0.48
**XGI-198**	RMSF (Å) over RBD 330-530	0.79 ± 0.06	0.81 ± 0.05	0.28
	Number of inter-protein H-bonds	13.5 ± 1.0	13.2 ± 1.1	0.55
	Crystal contacts retained (%)	87 ± 3	86 ± 3	0.52
	Complex RMSD after 200 ns (Å)	1.8 ± 0.2	1.9 ± 0.2	0.39
**XGI-203**	RMSF (Å) over RBD 330-530	0.85 ± 0.04	0.83 ± 0.05	0.35
	Number of inter-protein H-bonds	12.8 ± 0.9	12.6 ± 1.0	0.63
	Crystal contacts retained (%)	85 ± 4	86 ± 3	0.57
	Complex RMSD after 200 ns (Å)	2.0 ± 0.2	2.1 ± 0.2	0.44

All replicates used identical simulation parameters and were performed on the same hardware. Note that p-values > 0.05 indicate no statistically significant difference between the original single trajectory and the replicate average.
